# Wearable Sensor Technologies and Gait Analysis for Early Detection of Dementia: Trends and Future Directions

**DOI:** 10.3390/s25247669

**Published:** 2025-12-18

**Authors:** Anna Tsiakiri, Spyridon Plakias, Georgios Giarmatzis, Georgia Tsakni, Foteini Christidi, Georgia Karakitsiou, Vasiliki Georgousopoulou, Georgios Manomenidis, Dimitrios Tsiptsios, Konstantinos Vadikolias, Nikolaos Aggelousis, Pinelopi Vlotinou

**Affiliations:** 1 Department of Neurology, Democritus University of Thrace, 68100 Alexandroupolis, Greece; christidi.f.a@gmail.com (F.C.); kvadikol@med.duth.gr (K.V.); 2 Department of Physical Education and Sport Science, University of Thessaly, 42100 Trikala, Greece; spyros_plakias@yahoo.gr; 3 Department of Physical Education and Sport Science, Democritus University of Thrace, 69100 Komotini, Greece; ggiarmat@phyed.duth.gr (G.G.); nagelous@phyed.duth.gr (N.A.); 4 Department of Occupational Therapy, University of West Attica, 12243 Athens, Greece; ytsakni@uniwa.gr (G.T.); pvlotinou@uniwa.gr (P.V.); 5 Department of Psychiatry, Medical School, Democritus University of Thrace, 68100 Alexandroupolis, Greece; gkarakit@med.duth.gr; 6 Department of Nursing, Democritus University of Thrace, 68100 Alexandroupolis, Greece; vageorgo@nurs.duth.gr (V.G.); gmanomen@nurs.duth.gr (G.M.); 7 3rd Department of Neurology, Aristotle University of Thessaloniki, 54124 Thessaloniki, Greece; tsiptsios.dimitrios@yahoo.gr

**Keywords:** gait analysis, wearable sensors, dementia, mild cognitive impairment, early diagnosis, digital biomarkers

## Abstract

The progressive nature of dementia necessitates early detection strategies capable of identifying preclinical cognitive decline. Gait disturbances, mediated by higher-order cognitive functions, have emerged as potential digital biomarkers in this context. This bibliometric review systematically maps the scientific output from 2010 to 2025 on the application of wearable sensor technologies and gait analysis in the early diagnosis of dementia. A targeted search of the Scopus database yielded 126 peer-reviewed studies, which were analyzed using VOSviewer for performance metrics, co-authorship networks, bibliographic coupling, co-citation, and keyword co-occurrence. The findings delineate a multidisciplinary research landscape, with major contributions spanning neurology, geriatrics, biomedical engineering, and computational sciences. Four principal thematic clusters were identified: (1) Cognitive and Clinical Aspects of Dementia, (2) Physical Activity and Mobility in Older Adults, (3) Technological and Analytical Approaches to Gait and Frailty and (4) Aging, Cognitive Decline, and Emerging Technologies. Despite the proliferation of research, significant gaps persist in longitudinal validation, methodological standardization, and integration into clinical workflows. This review emphasizes the potential of sensor-derived gait metrics to augment early diagnostic protocols and advocates for interdisciplinary collaboration to advance scalable, non-invasive diagnostic solutions for neurodegenerative diseases.

## 1. Introduction

Dementia, with Alzheimer’s disease (AD) as its most prevalent form, represents one of the most pressing public health challenges of the 21st century. Currently, over 55 million people globally are living with dementia—a number projected to soar to 139 million by 2050 due to aging populations, particularly in low- and middle-income countries where the sharpest increases are anticipated [1]. AD alone accounts for 50–70% of dementia cases and imposes a profound and multifaceted burden on individuals, families, health systems, and economies worldwide [2]. The global cost of dementia care was estimated at $1.3 trillion in 2019 and is expected to escalate to $1.7 trillion by 2030, with nearly half of this cost attributed to informal care provided by unpaid family members, most of whom are women [1]. Despite advances in understanding the disease, there is still no curative treatment, making prevention, early diagnosis, and integrated care essential policy priorities [3]. The World Health Organization (WHO) and major scientific bodies have recognized dementia as a global public health priority, emphasizing the urgent need for coordinated international strategies to reduce risk factors, support caregivers, and alleviate health disparities [4]. Around 40% of global dementia cases may be preventable through modification of key risk factors such as hypertension, smoking, obesity, physical inactivity, and social isolation—factors that disproportionately affect disadvantaged populations [3]. However, national policy responses remain inadequate, with only a quarter of countries having implemented dedicated dementia plans, many of which lack adequate funding or sustainability [1]. As the prevalence of dementia continues to rise, inaction risks overwhelming healthcare systems, exacerbating social inequities, and undermining the well-being of older adults globally. The growing burden of dementia calls for immediate public health action grounded in scientific evidence, social justice, and economic foresight.

Early diagnosis of dementia is a critical public health imperative with significant implications for individual well-being, health care systems, and societal burden. Despite increasing awareness, it is estimated that up to 60% of dementia cases remain undiagnosed in primary care settings, thereby delaying access to vital resources and interventions [5]. As dementia progresses gradually, early identification—especially at the stage of mild cognitive impairment (MCI)—enables clinicians to initiate timely interventions that may slow cognitive decline and optimize the patient’s remaining functional years [6,7,8]. Furthermore, early diagnosis empowers patients and caregivers to plan for future care needs, make informed decisions about legal and financial matters, and access support services that improve quality of life [6,9]. From a systemic perspective, delaying the onset or progression of dementia through early detection could result in substantial cost savings and a reduced strain on long-term care services [10]. Despite these benefits, multiple barriers, such as stigma, limited training in primary care, and disparities in access among rural and minority populations, continue to hinder widespread implementation of early detection strategies. Therefore, enhancing clinical protocols, policy support, and public awareness around early diagnosis is essential to reduce the burden of dementia and improve outcomes for affected individuals and their families.

The integration of gait analysis and wearable sensor technologies is increasingly recognized as a promising avenue for the early detection and monitoring of cognitive impairment, including dementia. Gait, a complex motor function governed by cognitive processes such as attention, executive functioning, and memory, often deteriorates subtly during the early stages of cognitive decline, making it a sensitive behavioral biomarker [11,12,13]. Recent studies have demonstrated that individuals with amnestic mild cognitive impairment (aMCI), a precursor to Alzheimer’s disease, exhibit significant abnormalities in gait parameters, including reduced walking velocity, shorter stride length, and increased stride time variability compared to cognitively healthy controls [14]. These abnormalities correlate with specific domains of cognitive decline, particularly memory and executive function, supporting the view that motor and cognitive deficits share underlying neural pathways. Traditional diagnostic tools such as neuroimaging and cerebrospinal fluid biomarkers, although effective, are expensive, invasive, and often inaccessible in primary care or low-resource settings. In contrast, gait analysis using wearable sensors or depth cameras like Kinect v.2 offers a cost-effective, non-invasive, and scalable solution [12]. Wearable devices enable continuous, real-world monitoring of spatiotemporal gait parameters, which may better reflect functional impairments in daily life compared to laboratory-based assessments [14]. Furthermore, machine learning algorithms applied to gait data have shown high accuracy in distinguishing individuals with MCI from healthy controls, particularly when analyzing more cognitively demanding walking tasks like oval-path walking [12]. This convergence of digital health technologies, sensor data, and artificial intelligence not only enhances diagnostic precision but also holds the potential to transform routine clinical assessments by enabling earlier, more personalized interventions for individuals at risk of dementia.

Despite the growing body of evidence supporting gait analysis and wearable sensors as valuable tools in the detection of cognitive decline, the field still lacks systematic bibliometric mapping that could consolidate existing knowledge and guide future research directions. While numerous studies have independently highlighted the diagnostic potential of gait-related biomarkers, there is a notable absence of comprehensive, data-driven analyses that chart trends, influential publications, collaborative networks, and emerging hotspots in this interdisciplinary field. As recently noted by Zhong et al. [15], the research landscape on gait analysis in older adults with MCI remains fragmented and underexplored from a bibliometric perspective, hindering the development of a unified scientific framework. The lack of such systematic reviews and knowledge maps limits researchers’ ability to avoid redundancy, build on existing evidence, and strategically prioritize innovative approaches that could accelerate early diagnosis of dementia. Therefore, bibliometric mapping should be considered a critical component of advancing the field, promoting interdisciplinarity, and enhancing both the scientific and clinical impact of gait-based cognitive assessments.

This study aims to provide a focused bibliometric analysis of peer-reviewed journal articles published between 2010 and 2025 that examine the role of gait analysis and wearable sensor technologies in the early diagnosis or detection of dementia and Alzheimer’s disease. Despite increasing interest in this interdisciplinary field, no prior study has systematically mapped the scientific output using this combination of clinical, biomechanical, and technological criteria. The goal is to offer a structured overview of the field and to support future interdisciplinary research aimed at improving early detection and intervention strategies through non-invasive, sensor-based gait assessment.

## 2. Materials and Methods

### 2.1. Search Strategy

A targeted literature search was conducted in Scopus database to identify peer-reviewed articles related to the use of gait analysis, motion capture, and biomechanics in the early detection and diagnosis of dementia, with particular emphasis on Alzheimer’s disease. The search aimed to capture studies that explore the application of wearable technologies or sensors for assessing movement-related biomarkers in human subjects.

The following search query was used:

TITLE-ABS-KEY ((“dementia” OR “Alzheimer” OR “mild cognitive impairment” OR “cognitive decline”) AND (“early diagnosis” OR “early detection” OR “screening” OR “biomarker” OR “predict” OR “prognos” OR “monitoring” OR “assessment”) AND (“gait” OR “gait analysis” OR “walking” OR “locomotion” OR “mobility” OR “motion capture” OR “movement analysis” OR “biomechanics” OR “motor function”) AND (“wearable sensor” OR “inertial measurement unit” OR “IMU” OR “acceleromet” OR “gyroscope” OR “wearable device” OR “sensor” OR “portable device”)) AND PUBYEAR > 2009 AND PUBYEAR < 2026 AND (LIMIT-TO (DOCTYPE, “ar”)) AND (LIMIT-TO (LANGUAGE, “English”)) AND (LIMIT-TO (EXACTKEYWORD, “Humans”)).

The search was limited to articles published in English between 2010 and 2025 and included only original research involving human participants. Studies that met the criteria were drawn from reputable scientific databases, ensuring relevance to both clinical and technological aspects of dementia research. The goal was to highlight recent advances in wearable-assisted movement analysis as a tool for detecting cognitive decline at early stages.

### 2.2. Selection Criteria

Studies were selected according to predefined inclusion and exclusion criteria, with the aim of identifying research that aligns with the study objective: the use of gait-related or biomechanical markers, obtained through wearable technologies, for the early detection or diagnosis of dementia in human populations. The inclusion criteria required that studies: (i) be original research articles published in peer-reviewed journals, (ii) involve human participants, and (iii) focus on the application of gait analysis, motion capture, or biomechanical methods in the context of early-stage dementia or Alzheimer’s disease, utilizing wearable devices or sensors.

The exclusion criteria were applied to ensure the relevance and scientific rigor of the included literature. Studies were excluded if they: (i) involved non-human subjects, particularly animal models such as rats; (ii) were case reports, which often lack generalizability and statistical validity; or (iii) were protocol papers, which describe intended methodologies but do not report empirical findings. These selection criteria were applied during the screening and eligibility assessment phases of the review to ensure that only studies with direct applicability to human-centered, technology-driven approaches for early dementia detection were retained for analysis.

### 2.3. Data Extraction

Bibliographic data for all articles meeting the inclusion criteria were retrieved directly from the Scopus database. The export was conducted in CSV format, which included detailed metadata for each publication. Specifically, the dataset comprised information on article title, list of authors, author affiliations, journal of publication, year of publication, abstract, number of citations, and assigned keywords. This structured format allowed for a comprehensive overview of the publication characteristics relevant to the scope of this study. It should be noted that funding information was not included in the export, as this field is not available by default in the Scopus CSV output.

Following data export, the file was imported into VOSviewer (version 1.6.20.0), a widely used open-source tool for constructing and visualizing bibliometric networks [16,17]. The software was employed to perform bibliometric mapping and co-occurrence analysis of author keywords and terms extracted from abstracts. These analyses facilitated the identification of thematic clusters, emerging trends, and interrelationships among key concepts within the literature. This methodological step supported both the descriptive characterization of the research landscape and the quantitative exploration of conceptual linkages in the field of early dementia detection using gait analysis and wearable technologies.

### 2.4. Bibliometric Analysis

The bibliometric analysis in this study combined performance analysis and science mapping techniques, following established methodological standards in bibliometric research [18,19,20]. All analyses were conducted using VOSviewer (version 1.6.20.0), a specialized software tool for constructing and visualizing bibliometric networks. Prior to analysis, preprocessing procedures were implemented to enhance data consistency, including the use of custom thesaurus files to correct for variations and spelling inconsistencies in author names, journal titles, and keywords.

#### 2.4.1. Performance Analysis

The performance analysis aimed to quantify research productivity and influence across both individual and source-level contributions. In the author productivity analysis, only authors with a minimum of four publications were included. The size of each node in the visualizations represented the total number of documents contributed by each author. Similarly, the source analysis evaluated journal-level productivity, applying a threshold of three documents per source, with node weights again determined by document count. Summary tables and network diagrams were generated to highlight the most prolific authors and publication venues within the dataset.

#### 2.4.2. Science Mapping Analysis

Science mapping techniques were employed to uncover structural, thematic, and collaborative patterns within the literature. Four distinct approaches were used:

- Co-authorship Analysis: This analysis focused on institutional affiliations, treating organizations as the unit of analysis. Institutions with at least two published documents were included. The resulting network visualized collaboration patterns between institutions, with node sizes reflecting the number of documents associated with each organization.
- Bibliographic Coupling: Journals were used as the unit of analysis to assess thematic similarity based on shared references. A minimum of two documents per source was required for inclusion. The resulting overlay visualization identified clusters of journals with overlapping citation profiles.
- Co-citation Analysis: This technique was used to identify the intellectual structure and key scholarly influences within the field. It focuses on detecting sources (i.e., journals) that are frequently cited together across different publications, which may indicate shared conceptual backgrounds or thematic alignment. Τhe unit of analysis was the source (journal), and a minimum threshold of 10 co-citations was applied to ensure relevance and statistical robustness. This mapping approach enables the identification of influential publications and intellectual schools of thought [8,16].
- Keyword Co-occurrence Analysis: Author keywords served as the unit of analysis to explore recurring themes and emerging trends. Keywords that appeared at least three times across the corpus were included. Co-occurrence patterns were visualized as a network, with node size weighted by keyword frequency. Clustering was automatically performed by VOSviewer based on link strength and proximity, revealing major thematic groupings in the field.

A detailed summary of the methodological settings applied in each science mapping technique is provided in Table 1.

## 3. Results

### 3.1. Included Studies

A total of 184 records were initially retrieved from the Scopus database following the application of the search strategy described in Section 2.1. These records were screened based on predefined inclusion and exclusion criteria (see Section 2.2). After removing studies that did not meet the eligibility requirements, 126 articles were included (Table S1). 

### 3.2. Bibliometric Performance Analysis

The performance analysis focused on identifying the most prolific contributors in the field by examining author productivity, using the number of published documents as the primary metric. Authors who had contributed to at least three publications within the final set of included studies were retained for analysis. A total of 25 authors met this threshold. Table 2 summarizes the bibliometric performance of the most productive and influential authors within the analyzed research domain. The data include the number of publications and corresponding citation counts, reflecting both research productivity and scholarly impact. Among the listed authors, Kaye, Jeffrey A. exhibits the highest citation record (563 citations from 6 documents), indicating a strong academic influence. Other authors with notable citation performance include Mattek, Nora C. (426 citations), Hayes, Tamara L. (407 citations), and Najafi, Bijan (289 citations). The overall distribution reveals a concentrated contribution pattern, wherein a limited number of authors account for a substantial proportion of citations, underscoring their pivotal roles in shaping the intellectual structure of the field.

**Figure 1:**
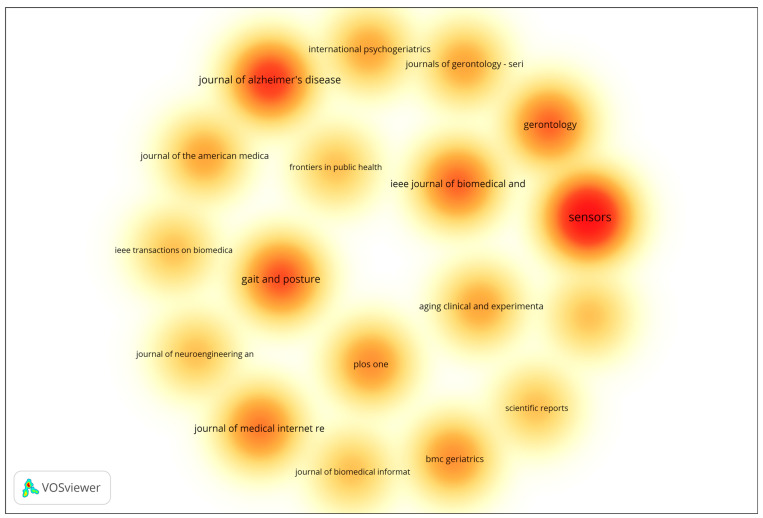
Density Visualization of Most Productive Journals. The density map uses a color gradient to represent the concentration of publications across journals. Warmer colors (yellow → orange → red) indicate higher publication density and greater research activity, with red areas representing the most productive journals. Cooler and lighter colors indicate lower publication density and fewer contributions.

In addition to author-level productivity, source-level performance was also analyzed to identify the most prominent journals publishing research on gait analysis and wearable technologies for early dementia detection. The analysis included all sources with a minimum of two documents, using the number of documents as the weighting metric. The resulting density visualization (Figure 1) highlights clusters of influential publication venues based on document frequency. The visualization highlights the density and frequency of publications across various scientific outlets. Journals represented by warmer colors (red and orange) correspond to higher publication activity, indicating their prominence within the field. The results reveal that Sensors, Journal of Alzheimer’s Disease, and Gait and Posture are among the most influential publication venues, characterized by their high concentration of documents and strong citation performance. Other journals such as IEEE Journal of Biomedical and Health Informatics, Gerontology, and Journal of Medical Internet Research also demonstrate considerable research activity, underscoring the multidisciplinary nature of the field, which spans biomedical engineering, gerontology, and digital health. Overall, the overlay visualization underscores the dominance of a select group of specialized journals that serve as key dissemination platforms for research on aging, mobility, and sensor-based health monitoring. These outlets play a pivotal role in advancing interdisciplinary collaboration and the translation of technological innovations into clinical and public health applications.

### 3.3. Science Mapping

#### 3.3.1. Co-Authorship Analysis by Country

**Figure 2:**
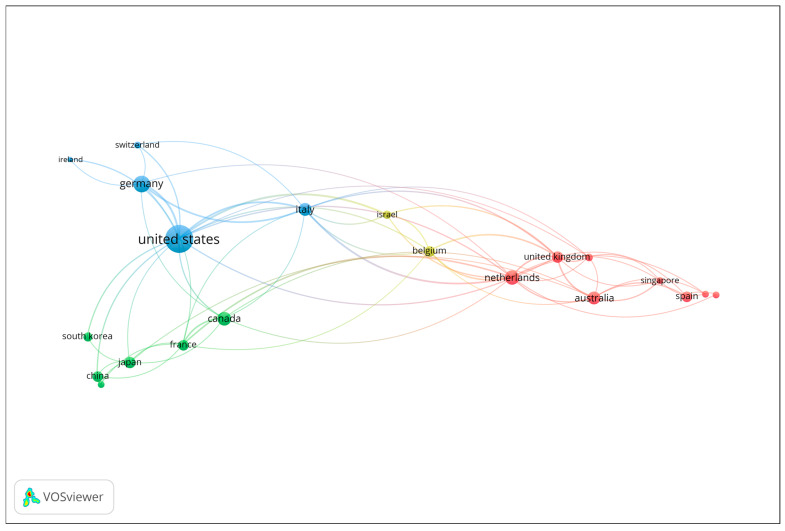
Co-authorship network of contributing countries (Minimum 2 publications; Node size = number of documents).

Co-authorship analysis at the country level was performed to explore patterns of international collaboration in the field of gait analysis and wearable technologies for early dementia detection. Countries contributing to at least two publications were included in the analysis. The number of documents served as the weighting metric, and the resulting network visualization is presented in Figure 2. Each node represents a country, with node size corresponding to the number of publications, and the connecting lines (links) indicating collaborative relationships based on co-authored documents. The color coding reflects distinct collaboration clusters, which highlight regional and transnational research partnerships. The visualization reveals that the United States occupies a central and dominant position within the network, demonstrating extensive collaborative links with several European and Asian countries. Strong research connections are observed between the United States, the United Kingdom, Italy, Germany, and the Netherlands, forming a core collaborative cluster that drives much of the scholarly output in the field. Other notable contributors, including Canada, Australia, and Japan, also exhibit active engagement within this network, though to a lesser extent. Overall, the co-authorship network underscores the highly collaborative and international nature of research in this domain, with a concentration of productivity and influence in a few key countries that serve as major hubs of scientific exchange and interdisciplinary innovation.

#### 3.3.2. Bibliographic Coupling Analysis of Organizations

**Figure 3:**
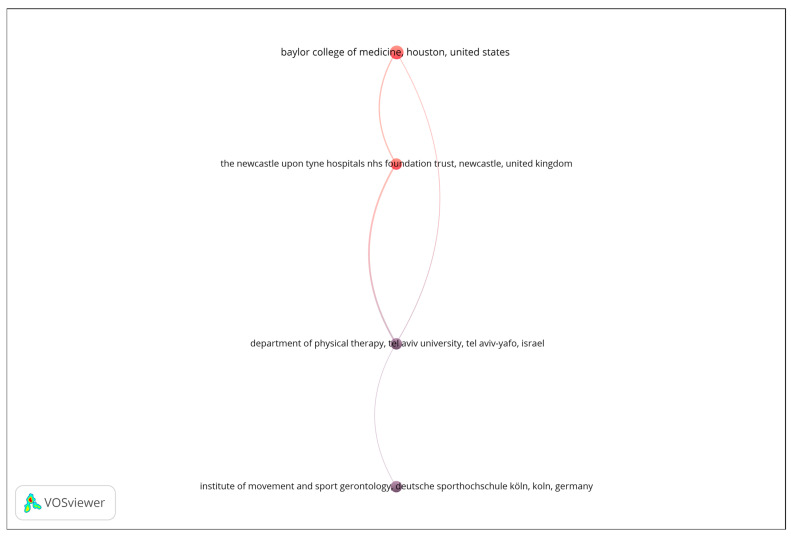
Bibliographic coupling network of organizations.

A bibliographic coupling analysis was performed to explore thematic similarities among publication venues based on shared reference patterns. The analysis was restricted to organizations with a minimum of four documents, and the number of documents was used as the weighting metric. As shown in Figure 3, the resulting network reveals a limited number of interconnected institutions, suggesting focused but relatively narrow collaboration patterns in the analyzed research domain. The visualization displays four nodes linked through bibliographic coupling relationships, forming a single, vertically oriented structure that represents distinct yet thematically aligned research entities. The strength of the coupling among these institutions indicates shared research interests and overlapping citation behavior, reflecting a convergence around specific topics such as biomedical engineering, sensor technologies, and aging-related health monitoring. Overall, the bibliographic coupling map underscores the thematic coherence of the leading organizations contributing to this field, while also highlighting the opportunity for broader institutional collaboration to enhance knowledge exchange and interdisciplinary integration.

#### 3.3.3. Co-Citation Analysis

**Figure 4:**
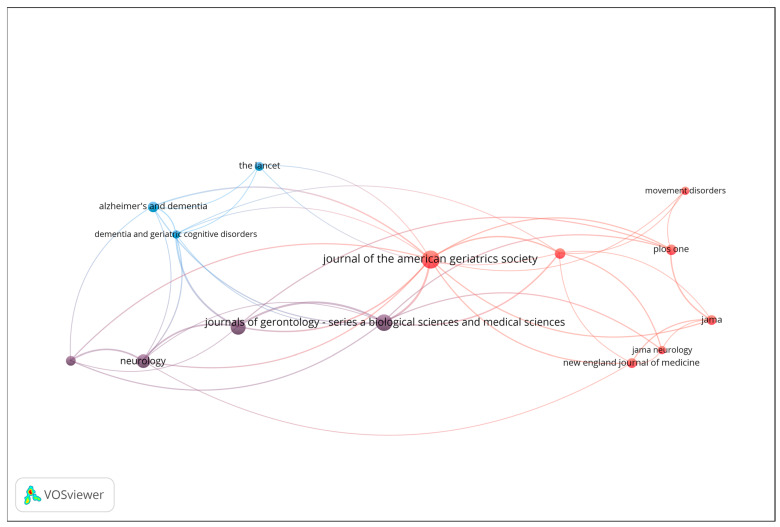
Co-citation network of influential sources (Minimum 6 citations; Node size = number of co-citations).

Co-citation analysis was conducted to uncover the intellectual structure and influential sources within the field of gait analysis and wearable sensor technologies for early dementia detection. This technique identifies journals and articles that are frequently cited together, reflecting shared conceptual foundations or complementary scientific contributions. Figure 4 presents the resulting co-citation network, including only sources with a minimum of 6 citations. The size of each node represents the total number of co-citations, while the proximity and thickness of connecting lines indicate the strength of co-citation relationships.

The red cluster, located at the center-right of the map, represents the clinical and neurology-oriented core of the field. It includes highly influential journals such as *Journal of the American Geriatrics Society (JAGS)*, *JAMA Neurology*, *The New England Journal of Medicine (NEJM)*, *JAMA*, *PLOS ONE*, and *Movement Disorders*. This cluster signifies a strong concentration of general medical and neurological research, highlighting journals that serve as hubs for interdisciplinary clinical studies. The blue cluster, positioned toward the left of the visualization, is associated with dementia and geriatrics research. It comprises journals such as *The Lancet, Alzheimer’s & Dementia*, and *Dementia and Geriatric Cognitive Disorders*. These titles form a cohesive subfield focused on neurodegenerative diseases and aging-related health, while maintaining strong citation links to broader clinical outlets, particularly those in the red cluster. The purple cluster, situated between the blue and red regions, acts as a bridge between neurology and gerontology. It includes *Neurology* and the *Journal of Gerontology: Series A (Biological Sciences & Medical Sciences)*. This cluster connects basic research on aging and neurobiology with clinical and population-level studies, thereby linking specialized and general medical discourses.

Overall, the co-citation analysis reveals a clear intellectual gradient extending from dementia and geriatrics (blue) through neurology and aging sciences (purple) to the general and clinical medicine core (red). This structure underscores the multidisciplinary nature of the field, where clinical geriatrics, neurological disorders, and aging research converge through shared citation patterns and overlapping thematic concerns.

#### 3.3.4. Co-Occurrence Analysis of Author Keywords

**Figure 5:**
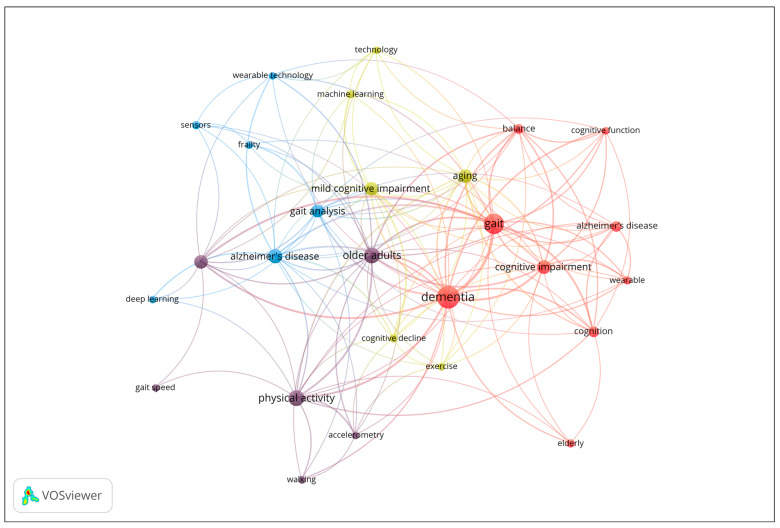
Co-occurrence network of author keywords (Minimum 4 occurrences; Node size = frequency of occurrence).

To investigate the thematic structure of the research field, a co-occurrence analysis of author keywords was performed. Keywords with a minimum occurrence of four were included in the analysis, and occurrence frequency was used as the weight metric. The results are displayed in the network visualization in Figure 5, where node size corresponds to the frequency of keyword use, and clustering is determined by co-occurrence strength. The network reveals four main clusters representing distinct but interrelated research themes. The map reveals four distinct thematic clusters. Cluster 1 (red) focuses on cognitive and clinical aspects of dementia, including keywords such as Alzheimer’s disease, cognition, dementia, and gait. Cluster 2 (purple) centers on physical activity and mobility in older adults, with terms like accelerometer, gait speed, and walking. Cluster 3 (blue) represents technological and analytical approaches, including deep learning, sensors, and wearable technology. Finally, Cluster 4 (yellow) highlights aging, cognitive decline, and emerging technologies, with keywords such as machine learning, exercise, and mild cognitive impairment. The network illustrates the interdisciplinary nature of the field, linking clinical, cognitive, and technological research domains in aging and dementia studies.

From the co-occurrence analysis of author keywords in the included publications, three main thematic clusters emerged, reflecting the interdisciplinary nature of research on dementia and gait analysis:

Cluster 1. Cognitive and Clinical Aspects of Dementia (9 items): Alzheimer’s disease, Balance, Cognition, Cognitive function, Cognitive impairment, Dementia, Elderly, Gait, Wearable [21,22,23,24,25,26,27,28,29,30,31,32,33,34,35,36,37,38,39,40,41,42,43,44,45,46,47,48,49,50,51,52,53,54,55,56,57,58,59,60,61,62,63,64].
Cluster 2. Physical Activity and Mobility in Older Adults (6 items): Accelerometer, Accelerometry, Gait speed, Older adults, Physical activity, Walking [65,66,67,68,69,70,71,72,73,74,75,76,77,78,79].
Cluster 3. Technological and Analytical Approaches to Gait and Frailty (6 items): Alzheimer’s disease, Deep learning, Frailty, Gait analysis, Sensors [80,81,82,83,84,85,86,87,88,89,90,91,92,93,94,95,96,97,98,99,100,101,102,103,104,105,106,107,108,109,110,111]
Cluster 4. Aging, Cognitive Decline, and Emerging Technologies (6 items): Aging, Cognitive decline, Exercise, Machine learning, Mild cognitive impairment, Technology [112,113,114,115,116,117,118,119,120,121,122,123,124,125,126,127,128,129,130,131,132,133,134,135,136,137,138,139,140,141,142,143,144,145,146]

## 4. Discussion

### 4.1. Interpretation of Thematic Cluster 1

The first thematic cluster highlights the growing convergence between cognitive, motor, and clinical dimensions of dementia. Across the included studies, dementia emerges not as a purely cognitive disorder but as a multisystem condition, detectable through alterations in movement, rhythm, environmental exposure, and affective responses.

A recurring theme involves the integration of cognitive–motor assessments as sensitive biomarkers of early cognitive decline. Dual-task paradigms that couple cognitive and motor performance outperform traditional screening tools. Schiavo et al. [22] validated the Performance Index (P-Index) as a quantitative measure combining gait and cognitive accuracy during the instrumented Timed Up and Go test, strongly predicting MMSE outcomes. Similarly, Greene et al. [31] introduced the Dual Task Ball Balancing Test, a safe digital assessment linked with both MCI and AD related dementia, emphasizing the shift toward ecologically valid, sensor-based cognitive testing.

Motor parameters such as gait variability and balance serve as additional indicators of neurodegeneration. Labott et al. [23] showed higher minimum toe clearance variability in MCI, while Schmidt et al. [29] and Chiba et al. [32] found poorer balance and gait patterns predicting falls and wheelchair dependence. These findings, supported by Aznielle-Rodríguez et al. [33], confirm that spatiotemporal gait features reflect global cognitive status and can function as digital proxies for brain health. Beyond motor correlates, neurobiological and environmental determinants are increasingly recognized. Xiong et al. [21] linked individual exposure to particulate matter and volatile organic compounds with cerebrospinal tau and amyloid biomarkers, revealing potential environmental pathways in dementia pathogenesis. Likewise, Elasfar et al. [27] differentiated AD from dementia with Lewy bodies (DLB) through gait analysis, suggesting that movement alterations precede amyloid accumulation.

Advances in wearable and digital monitoring expand clinical observation into real-world settings. Chan et al. [24] demonstrated that wrist-worn sensor data on walking speed, running duration, and bedtime predicted incident dementia with accuracy comparable to risk scores, while Hammink et al. [26] used biometric sensors in nursing homes to associate emotional responses with daily activities, offering insights into lived experience and under-stimulation in dementia care. Finally, studies like Slusarenko et al. [25] illuminate the embodied and rhythmic aspects of cognition. They showed that rhythmic proficiency and musical engagement enhance motor performance in healthy adults but not in MCI, implying that cognitive impairment disrupts rhythm-based coordination—yet such therapies retain rehabilitative potential.

In synthesis, this cluster portrays dementia as a complex interaction of cognitive, motor, emotional, and environmental factors. The convergence of dual-task testing, digital gait metrics, and ecological monitoring supports a personalized, sensor-informed approach to diagnosis and care. Individualized assessment whether through environmental exposure, mobility behavior, or affective response constitutes the foundation for next-generation dementia management.

### 4.2. Interpretation of Thematic Cluster 2

This cluster emphasizes how physical activity and mobility serve as core indicators of healthy aging, linking movement patterns with physical, cognitive, and functional outcomes. The integration of wearable technologies and objective accelerometry has transformed our understanding of mobility from a descriptive behavior to a quantifiable biomarker of aging health.

Evidence consistently shows that quality, not just quantity, of movement matters most. Continuous walking in longer bouts and higher cadence patterns have been associated with reduced care service use and lower risk of cognitive impairment, even after adjusting for age and comorbidities [66,67]. These findings suggest that mobility rhythm and endurance provide early insight into overall resilience and independence. Physiological analyses further support this link: moderate-to-vigorous activity enhances skeletal muscle energetics [70], while light daily activity improves short-term memory and inhibition among older adults with chronic illness [74].

The technological evolution of wearables enables continuous, non-invasive monitoring of health behaviors. Reviews and bibliometric analyses reveal rapid growth in smart wearable applications for fall detection, disease prevention, and mobility tracking [65,68]. When combined with machine learning, sensor data can predict functional decline and physical impairment with high precision [69]. Importantly, studies stress the need for user-centered design and long-term validation to ensure reliability in older populations. Mobility monitoring also plays a preventive role in fall and dependency prediction. Gait quality and walking speed derived from wrist-worn devices predict injurious falls [72], while sensor-based activity patterns outperform traditional tests in identifying fall risk among people with dementia [79]. Even in the oldest-old, higher activity intensity relates to better nutrition and motor performance, whereas sedentary behavior in institutional care remains a persistent challenge [71,76,78].

Overall, the cluster reveals a paradigm shift toward data-driven mobility assessment. Physical activity emerges as a multidimensional biomarker connecting musculoskeletal, cognitive, and psychosocial domains. Sustained, achievable levels of activity supported by wearable monitoring may thus represent one of the most practical strategies for maintaining autonomy and health in older adulthood.

### 4.3. Interpretation of Thematic Cluster 3

This cluster illustrates the rapid convergence of sensor technologies and artificial intelligence in analyzing gait and frailty among older and neurologically impaired adults. Collectively, these studies show how digital and analytical innovations are transforming movement assessment from clinical observation to continuous, data-driven monitoring.

A primary theme is the digitalization of gait assessment. Automated tools such as the electronic Short Physical Performance Battery (eSPPB) kiosk have demonstrated reliable, scalable frailty screening in community settings [80]. Similarly, ear-worn and depth-camera systems provide accurate gait speed and step length estimates, extending precision analysis beyond laboratories [87,91]. These advances make remote, objective mobility evaluation feasible in daily environments.

A second trend involves machine learning–based analytics capable of recognizing complex gait signatures. Deep learning models, such as LSTM and Enhanced Band-Dependent Learning, have achieved accurate classification of gait cycles and the detection of disease-related abnormalities, including Alzheimer’s-linked movement changes [81,85]. Such models move gait research toward predictive and diagnostic applications, offering real-time interpretation of sensor data.

At the same time, digital biomarkers derived from wearable devices are gaining clinical utility. Studies using explainable AI and quantile regression [88] improve the precision of gait feature extraction, while multicenter trials confirm their ability to distinguish neurological subtypes such as Alzheimer’s and Lewy body dementia [82]. Platforms like FACET further demonstrate that integrated home-based monitoring can reduce frailty progression [86].

Across this research, the fusion of multimodal sensing and analytical intelligence defines a shift toward precision gerontechnology. Inertial, radar, and vision-based systems now operate synergistically, offering multidimensional and longitudinal perspectives of human movement. These innovations position gait and frailty as dynamic, quantifiable, and modifiable health indicators, central to early detection and personalized intervention.

### 4.4. Interpretation of Thematic Cluster 4

This cluster highlights the rapid integration of digital innovation into aging and dementia research, where wearable sensors, smart-home systems, and artificial intelligence provide new ways to detect, monitor, and manage cognitive decline. The studies collectively show that behavioral and physiological data gathered unobtrusively can serve as early digital biomarkers of neurodegenerative change.

A dominant theme concerns AI-assisted diagnostics. Machine learning models using actigraphy and motor data have achieved high accuracy in distinguishing Alzheimer’s disease from other dementias and in detecting mild cognitive impairment [112,116,129]. Movement complexity, gait variability, and circadian rhythm metrics emerge as sensitive predictors of preclinical decline. These methods illustrate how everyday activity can act as a continuous reflection of cognitive health.

Equally important is the use of connected technologies for prevention and intervention. Telehealth and mHealth systems combine behavioral feedback with real-time monitoring. Tai Chi programs supported by wearables improved cognitive performance and sleep in diabetic older adults [114], while passive sensing interventions like Sense4Safety proved feasible for fall prevention in older adults with mild cognitive impairment [113]. Such models mark a shift toward personalized, technology-supported aging care.

The cluster also includes smart-home and ambient sensing applications capable of tracking behavioral and psychological symptoms of dementia [115,118]. These unobtrusive systems detect deviations in daily routines, while digital biomarkers have been correlated with Alzheimer’s pathology postmortem [119], underscoring their biological relevance. Moreover, longitudinal data link poor sleep and low physical activity with cognitive decline [120], and web-based multidomain programs demonstrate the feasibility of online cognitive and lifestyle interventions [128].

Overall, this cluster reveals a paradigm shift toward continuous, data-driven aging research, where cognition, mobility, and lifestyle are monitored dynamically. Emerging technologies not only enhance early detection but also support individualized prevention and care, transforming the traditional model of aging assessment into a proactive and personalized digital health framework.

**Figure 6:**
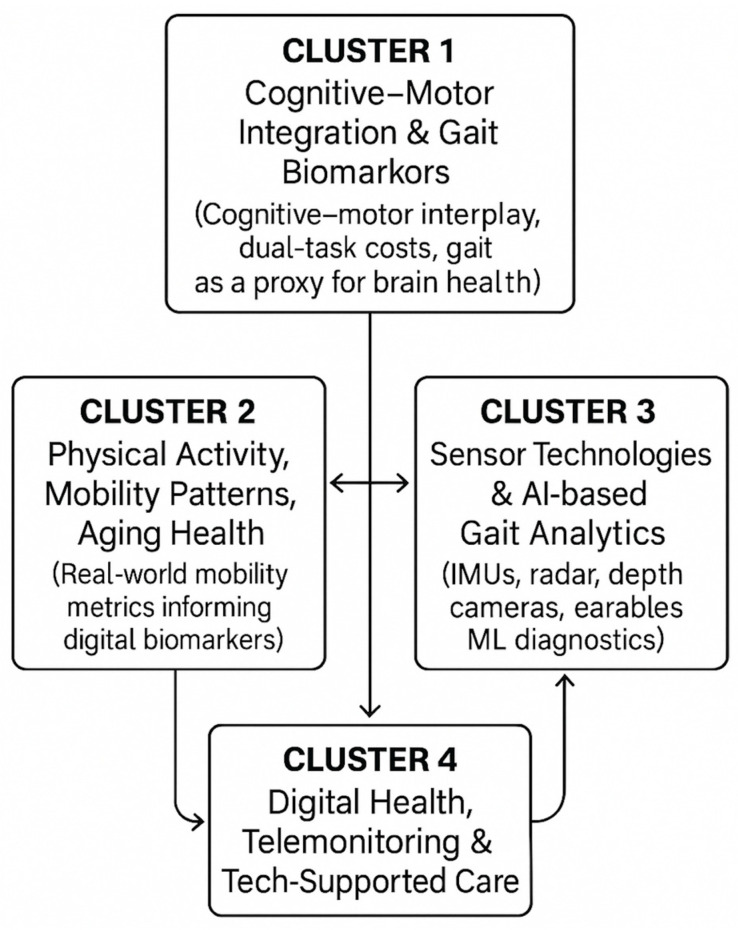
A Cluster Relationship Map.

Figure 6 visually depicts the interactions among the four thematic clusters (cognitive–motor integration, physical activity and mobility patterns, sensor/AI-driven gait analytics, and digital-health interventions). This figure highlights how the clusters connect conceptually (e.g., cognitive–motor biomarkers linking to gait features; sensor innovations linking to real-world mobility monitoring) and clarifies the overarching narrative of technological and clinical convergence in the field.

### 4.5. Causal Relationships and Neural Mechanisms Linking AD Pathology to Gait Abnormalities

**Figure 7:**
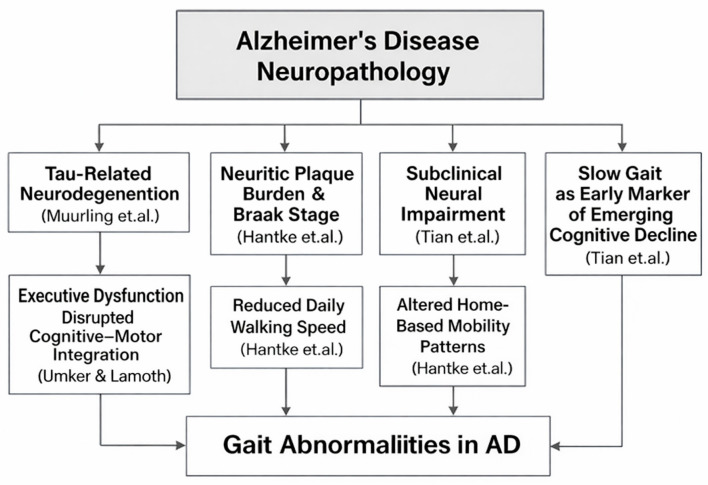
Mechanistic Pathways Linking AD Neuropathology to Alterations in Gait Parameters. The figure summarizes findings from studies on tau-related neurodegeneration (Muurling et al. [52]), neuritic plaque burden and Braak stage (Hantke et al. [119]), subclinical neural impairment and slow gait as an early marker of cognitive decline (Tian et al. [45]), and executive dysfunction affecting cognitive–motor integration (IJmker & Lamoth [64]).

Growing evidence suggests that gait impairments in AD reflect underlying neurodegenerative processes rather than merely age-related motor slowing. Within the provided studies, several findings connect biomarkers of AD pathology, including tau-related neurodegeneration and neuritic plaque severity, to altered gait domains, such as pace, rhythm, or walking speed (Figure 7). These relationships support the hypothesis that gait serves as an externally measurable expression of central nervous system decline. Among the studies linking biological markers to gait, Muurling et al. [52] demonstrate that specific gait domains, particularly rhythm, correlate with cerebrospinal fluid (CSF) tau levels, especially under dual-task conditions. This relationship suggests a mechanistic pathway wherein tau-associated neuronal loss, especially in fronto-subcortical and motor-integration networks, disrupts the temporal regulation of gait. Because dual-task walking imposes cognitive-motor demands, the finding that rhythm disturbances magnify in this condition aligns with known impairments in executive control and attentional resource allocation in tau-driven AD pathology.

In contrast to tau effects, Elasfar et al. [27] report that regional amyloid-β (Aβ) deposition shows no significant association with gait outcomes. This supports the broader view that amyloid burden alone does not directly modulate motor coordination or locomotor timing. Instead, gait abnormalities appear to emerge more strongly from downstream neurodegenerative changes, including tau accumulation, synaptic dysfunction, and cortical/subcortical atrophy, rather than from amyloid deposition itself. Adding further support, Hantke et al. [119] demonstrate that postmortem neuropathological markers, including neuritic plaque (NP) severity and Braak stage, correlate with reduced walking speed and other digital mobility indicators captured through long-term in-home monitoring. These findings underscore a direct physiological coupling between progressive AD neuropathology and declines in everyday motor behavior, strengthening the mechanistic argument that gait slowing and disruption serve as measurable proxies of accumulating plaques and tangles.

The longitudinal study by Tian et al. [45] further supports the mechanistic link between neural degeneration and gait by showing that slower gait speed in older adults predicts later development of MCI/AD, particularly when slow gait is not accompanied by compensatory activity fragmentation. The interpretation offered is that persistent slow gait in the absence of behavioral adaptation may reflect subclinical neurological impairmen, likely including early tau-driven degeneration in cortical and subcortical regions that regulate motor function. Further mechanistic insight is provided by IJmker & Lamoth [64], who show that dementia patients exhibit irregular trunk acceleration patterns and instability, linked closely to deficits in executive function. Given that executive impairments in AD are strongly associated with frontal and parietal tau pathology, the study supports a causal chain: Tau-driven executive dysfunction → impaired motor planning and stability → increased gait irregularity.

### 4.6. Diagnostic Thresholds and Quantitative Gait Indicators for Early Cognitive Decline

To strengthen the quantitative dimension of gait-related findings, we additionally synthesized numerical differences in key gait parameters across the included studies. Older adults with MCI consistently demonstrated slower maximal walking speed, with reductions of approximately 0.25–0.35 m/s compared to healthy controls, and this decrease was strongly predictive of incident dementia in long-term follow-ups (32% reduced hazard per SD increase in speed). Gait variability measures showed robust discriminatory power: minimum toe clearance (MTC) variability was significantly higher in MCI (*p* = 0.016, d = 0.53), while increased step-time and swing-time variability differentiated dementia subtypes and reflected underlying motor-cognitive impairment. Dual-task paradigms further amplified these differences, with dual-task walking producing notable decreases in gait speed, stride length, mid-swing elevation, and increased double-limb support among individuals with cognitive impairment. Balance-related quantitative differences were also evident, with cognitively impaired older adults showing significantly higher sway velocity and path length (ηp^2^ = 0.144–0.190) during standing tasks (Table 3).

### 4.7. Emerging Trends

Recent research across the analyzed literature reveals a clear evolution toward continuous, data-driven, and personalized monitoring of aging, mobility, and cognitive decline. Advances in multimodal sensing, ranging from inertial [147] and radar-based systems to ear-worn and depth-camera devices, are enabling unobtrusive, real-world gait analysis that extends beyond traditional laboratory settings [78,85,89]. These innovations, supported by the growing use of artificial intelligence and deep learning frameworks, have demonstrated high precision in identifying disease-related gait abnormalities and early motor-cognitive changes associated with Alzheimer’s disease and mild cognitive impairment [148]. At the same time, novel analytical approaches such as explainable AI and quantile regression models enhance the interpretability and reliability of digital biomarkers derived from wearable sensors [86]. Collectively, these developments signify a paradigm shift from static, episodic clinical assessments to continuous digital phenotyping, where movement, behavior, and physiological signals act as dynamic indicators of health and disease progression.

Parallel to diagnostic advances, technology-supported interventions are increasingly emerging as tools for prevention and rehabilitation [149,150,151,152,153]. Integrated mobile and telehealth systems demonstrate the potential to reduce frailty progression and improve functional outcomes through personalized feedback loops [84,111]. Digital therapeutics such as wearable-assisted Tai Chi programs show measurable benefits in cognition and sleep quality among older adults [112], while passive sensing systems like Sense4Safety provide real-time fall prevention and behavioral monitoring for individuals with mild cognitive impairment [111]. Furthermore, smart-home and ambient sensing technologies allow continuous detection of behavioral and psychological symptoms of dementia, correlating digital biomarkers with neuropathological findings [113,117]. These approaches collectively reflect a broader transformation of geriatric and dementia care from reactive diagnosis to proactive, sensor-informed management. As emphasized by recent studies, ensuring user accessibility, privacy, and ethical design remains essential for the equitable deployment of such technologies [116,124,125].

### 4.8. Interdisciplinary Nature of the Field

The bibliometric and thematic analysis underscores the profoundly interdisciplinary character of research on gait analysis, wearable sensors, and early dementia detection. The field brings together diverse domains (clinical neuroscience, geriatrics, biomechanics, computer science, and data analytics) to create an integrated understanding of how motor and cognitive functions interact across the aging process [20,63,78,113]. This convergence reflects a shift from discipline-specific approaches to a system-oriented framework, where cognition, mobility, physiology, and environment are analyzed as interdependent components of brain health. Studies combining clinical gait metrics with advanced sensor technologies and artificial intelligence exemplify this integration, demonstrating how digital methods can bridge the gap between traditional neurology, behavioral science, and engineering [79,83,86].

The interdisciplinary nature of the field is also evident in its collaborative research networks. International co-authorship patterns reveal strong partnerships between clinicians, engineers, and data scientists across the United States, the United Kingdom, Germany, and the Netherlands, countries that serve as central hubs for innovation in digital aging research [Figure 2]. Such collaborations have enabled the development of shared methodologies, open-access datasets, and translational frameworks that accelerate progress from laboratory research to clinical application. Importantly, this cross-sector cooperation supports the creation of ecologically valid digital biomarkers capable of detecting early cognitive decline in real-world environments [84,111,112].

Ultimately, the interdisciplinarity of this field is not merely structural but conceptual. It redefines dementia research through the integration of clinical insight, technological precision, and behavioral context. The joint efforts of clinicians, computer scientists, and public health experts are paving the way for precision gerontechnology, an emerging paradigm in which artificial intelligence and sensor-based analytics inform early diagnosis, prevention, and personalized care. Sustaining this momentum will require continued cross-disciplinary collaboration, standardized data frameworks, and ethical design principles to ensure that innovation in digital health remains inclusive, transparent, and clinically meaningful [116,124,125].

### 4.9. Gaps in the Literature and Practical Implications for Clinical and Technological Research

Despite the growing body of evidence supporting the use of digital technologies in the early detection of cognitive decline, the literature still presents significant limitations that hinder both generalizability and real-world implementation. One major gap is the lack of longitudinal studies that evaluate the long-term validity and diagnostic precision of tools such as wearable devices and machine learning models in naturalistic settings. Most current studies rely on short-term data collection or controlled environments, which do not adequately reflect the complex and evolving nature of cognitive trajectories in aging populations. In addition, the literature reveals a conceptual and methodological fragmentation. While various studies successfully highlight individual digital biomarkers, such as gait variability, speech patterns, or sleep disturbances, there is a noticeable absence of integrative frameworks that combine these modalities into comprehensive diagnostic models. This fragmentation reduces the potential for multimodal analysis, which could enhance sensitivity to subtle and preclinical changes in cognitive function.

Another challenge lies in the limited diversity of study populations. Many datasets are drawn from homogeneous, high-income cohorts, thereby constraining the applicability of findings to broader, more diverse aging communities. Furthermore, ethical and privacy concerns related to the passive and continuous collection of behavioral data are often insufficiently addressed, despite the vulnerable status of the target population. These limitations carry important implications for both clinical practice and future technological development. Clinically, there is a growing need for tools that not only detect early signs of decline but also support individualized screening and intervention strategies. This presupposes close collaboration between clinicians, engineers, and data scientists to ensure that new technologies are both technically robust and aligned with user needs and clinical realities. Technologically, future tools must be designed with usability and accessibility in mind. Considering the sensory, motor, and cognitive challenges faced by older adults, systems must adopt human-centered design principles that facilitate long-term adherence and minimize user burden. Finally, broader access to open, multimodal datasets and standardized evaluation protocols would accelerate the development of clinically meaningful AI applications and foster replicable, interdisciplinary research.

### 4.10. Limitations of the Study

While this review offers a comprehensive exploration of the literature at the intersection of wearable technology, gait analysis, and early dementia detection, certain limitations must be acknowledged. First and foremost, the study was based solely on articles indexed in the Scopus database, which, although extensive, may not encompass relevant works from other platforms. This could have led to the exclusion of key contributions, particularly from technical or clinical subfields that use alternative publication channels. Another important constraint is the included research is based on short-term or cross-sectional designs conducted in controlled laboratory environments. These settings, while methodologically sound, do not always capture the complexity of real-world behavior or the long-term progression of cognitive decline in diverse, aging populations. The interpretability of results is also affected by variability in how gait features, sensor technologies, and cognitive assessments are measured across studies. This lack of standardization hampers direct comparison and synthesis. Moreover, although the analysis reveals rich thematic clustering, the reliance on automated bibliometric tools may oversimplify the nuanced overlap between different domains, particularly in such an interdisciplinary field. These limitations underscore the importance of ongoing interdisciplinary collaboration, methodological refinement, and inclusivity in future research efforts.

## 5. Conclusions

This bibliometric and thematic analysis provides a comprehensive overview of the evolving landscape of research on wearable sensor-based gait and behavioral analysis for the early detection of dementia and mild cognitive impairment. The findings highlight a dynamic and rapidly expanding field, characterized by increasing interdisciplinary collaboration across clinical neuroscience, biomedical engineering, data science, and gerontology. Importantly, the study underscores both the promise and complexity of using gait as a digital biomarker. On one hand, wearable sensors enable continuous, non-invasive monitoring of motor and behavioral indicators associated with cognitive decline. On the other, methodological heterogeneity and a lack of standardization pose challenges to comparability, reproducibility, and clinical adoption. As the global burden of dementia continues to rise, there is an urgent need for accessible, cost-effective, and ecologically valid tools to support early diagnosis and monitoring. This review advocates for future research that emphasizes longitudinal study designs, cross-cultural validation, ethical data governance, and the integration of sensor-based methods into routine care pathways. Strengthening collaboration between technologists, clinicians, and policy-makers will be essential to harness the full potential of wearable systems in addressing the pressing challenges of cognitive aging.

## Figures and Tables

**Table 1:** Overview of Science Mapping Techniques.

Technique	Unit of Analysis	Minimum Threshold	Weight (Node Size)	Visualization Type
Co-authorship	Countries	2 documents	Number of documents	Network
Bibliographic Coupling	Organizations	4 documents	Number of documents	Network
Co-citation	Sources	6 citations	Number of documents	Network
Co-occurrence	Author Keywords	6 occurrences	Keyword frequency	Network

**Table 2:** Most Productive Authors by Number of Documents and Citations.

No.	Author	Documents	Citations
1	Najafi, Bijan	8	289
2	Kaye, Jeffrey A.	6	563
3	Del Din, Silvia	5	197
4	Hausdorff, Jeffrey M.	5	170
5	Mattek, Nora C.	5	426
6	Rochester, Lynn	4	197
7	Brodie, Matthew Andrew D.	4	57
8	Fleiner, Tim	4	48
9	Häussermann, Peter	4	48
10	Lord, S. R.	4	57
11	Mohler, Jane	4	253
12	Zhou, He	4	124
13	Zijlstra, Weibren	4	48
14	Austin, Daniel	3	252
15	Chan, Lloyd L.Y.	3	27
16	Dodge, Hiroko Hayama	3	275
17	Hayes, Tamara L.	3	407
18	Kunik, Mark E.	3	102
19	Lamoth, Claudine C.J.	3	228
20	Mc Ardle, Riona	3	95
21	Naik, Anand Dinkar	3	80
22	Schwenk, Michael	3	175
23	Taati, Babak	3	195
24	Thomas, Alan Jeffrey	3	95
25	Toosizadeh, Nima	3	108

**Table 3:** Quantitative Differences in Key Gait Parameters Between MCI and Healthy Controls Across Included Studies.

Gait Parameter	Finding/Difference
Maximal walking speed [24]	Each SD increase → 32% lower hazard for incident dementia; MCI/AD show reduced speed vs. controls
Daily step counts [24]	Lower step counts in individuals who later developed dementia (30% decrease per SD)
Step-time variability [27]	Increased in DLB vs. AD and controls; higher variability indicates greater motor-cognitive impairment
Swing-time variability [27]	Significantly increased in DLB relative to AD and CU groups
Stride velocity/stride length [27]	Reduced in DLB and AD compared to controls
Minimum Toe Clearance (MTC) variability [23]	Significantly higher in MCI (*p* = 0.016, d = 0.53)
Mean MTC [23]	No significant difference between healthy and MCI (*p* = 0.980)
Dual-task gait speed [30]	Slower gait speed under dual-task vs. single-task; larger decrements in cognitive impairment
Dual-task stride length [30]	Shorter stride length in cognitive impairment during dual-task walking
Dual-task mid-swing elevation [30]	Reduced in cognitive impairment during dual-task
Double-limb support (% time) [30]	Increased in MCI and dementia, greater under dual-task
Sway velocity (balance tests) [29]	Higher sway velocity in cognitively impaired (ηp^2^ = 0.190, *p* < 0.001)
Sway path length [29]	Significantly higher in cognitive impairment (ηp^2^ = 0.144, *p* < 0.001)
Turning performance [82]	IMU-based turning detection remains accurate; AD individuals show slower turning compared to controls
Hip extensor angle [32]	Lower hip extension angle in dementia → associated with wheelchair dependence

## Data Availability

No new data were created.

## Supplementary Materials

The following supporting information can be downloaded at https://doi.org/10.5281/zenodo.17439813. Table S1: Overview of included studies.
